# The Active and Periactive Zone Organization and the Functional Properties of Small and Large Synapses

**DOI:** 10.3389/fnsyn.2016.00012

**Published:** 2016-05-24

**Authors:** Raquel Cano, Lucia Tabares

**Affiliations:** Department of Medical Physiology and Biophysics, School of Medicine, University of SevilleSeville, Spain

**Keywords:** active zone, neurotransmitter release, endocytosis, periactive zone, release sites

## Abstract

The arrival of an action potential (AP) at a synaptic terminal elicits highly synchronized quanta release. Repetitive APs produce successive synaptic vesicle (SV) fusions that require management of spent SV components in the presynaptic membrane with minimum disturbance of the secretory apparatus. To this end, the synaptic machinery is structured accordingly to the strength and the range of frequencies at which each particular synapse operates. This results in variations in the number and dimension of Active Zones (AZs), amount and distribution of SVs, and probably, in the primary endocytic mechanisms they use. Understanding better how these structural differences determine the functional response in each case has been a matter of long-term interest. Here we review the structural and functional properties of three distinct types of synapses: the neuromuscular junction (NMJ; a giant, highly reliable synapse that must exocytose a large number of quanta with each stimulus to guarantee excitation of the postsynaptic cell), the hippocampal excitatory small synapse (which most often has a single release site and a relatively small pool of vesicles), and the cerebellar mossy fiber-granule cell synapse (which possesses hundreds of release sites and is able to translocate, dock and prime vesicles at high speed). We will focus on how the release apparatus is organized in each case, the relative amount of vesicular membrane that needs to be accommodated within the periAZ upon stimulation, the different mechanisms for retrieving the excess of membrane and finally, how these factors may influence the functioning of the release sites.

## Organization of the Release Apparatus in Small and Large Synapses

**Figure 1:**
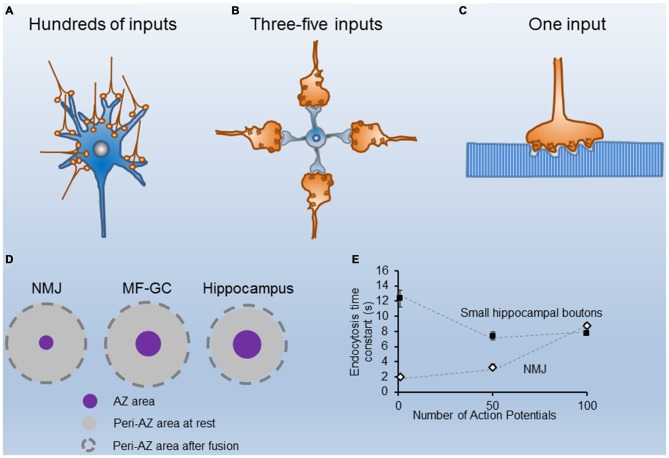
**Structural and functional properties of different nerve terminals. (A)** Cartoon of CA1 excitatory hippocampal boutons making contacts with a postsynaptic neuron. **(B)** Sketch of four cerebellar mossy fibers contacting a granule cell. **(C)** Illustration of a single motor nerve terminal innervating a muscle fiber. **(D)** Scaled representation of the mean sizes of single Active Zones (AZs; purple circles) and their corresponding peri-AZs (gray circles) in three synapse types (the neuromuscular junction (NMJ), the cerebellar mossy fiber-granule cell, and the hippocampus), at rest (gray circles), and after fusion of all synaptic vesicle (SVs) docked at each AZ (broken line circles). Note the similarity in the peri-AZ areas among different synapses. **(E)** Time constant of fluorescence recovery (tau) vs. number of stimuli (1, 50 and 100 AP) in mouse motor nerve terminals (white symbols adapted from Tabares et al., 2007; Gaffield et al., 2009), and in small hippocampal presynaptic boutons from neurons in culture, black symbols adapted from Armbruster et al. (2013).

Synaptic terminals differ in their strength and short-term plasticity, as well as in the size and spatial organization of the secretory apparatus, mainly in the dimensions, shape, and amount of their Active Zones (AZs), and in the size of their recycling pools of synaptic vesicles (SVs; Atwood and Karunanithi, 2002; Zhai and Bellen, 2004). Most terminals in the central nervous system (CNS) have a small number (1–8) of AZs, and the size of the recycling pool of SVs is not large. For instance, excitatory nerve terminals in area CA1 of the mouse hippocampus have a single AZ, about 10 docked vesicles per AZ, and a recycling pool of about 200 SVs (Schikorski and Stevens, 1997; Murthy et al., 2001; Rizzoli and Betz, 2005). Large terminals, on the other hand, such the rat cerebellar mossy fiber–granule cell synapse, the calyx of Held and the mouse neuromuscular junction (NMJ), have hundreds of AZs and a large recycling pool of SVs. These structural differences are in accordance with the specific functional roles of each synapse type. When the postsynaptic cell response is determined by the spatial and temporally integrated activity of hundreds of small terminals (Figure 1A), the number of quanta released per impulse (quantum content) per terminal is small. Conversely, when the postsynaptic cell receives information from only a few large nerve terminals, the sensitivity and fidelity of the transmission are usually very high. For example, in the cerebellar mossy fiber—granule cell synapse (Figure 1B), a burst of action potentials (APs) in a single mossy fiber bouton could be sufficient to generate spikes at the granule cell (Rancz et al., 2007). In this synapse, a large recycling pool of vesicles also contributes to sustaining transmission at high frequency (Saviane and Silver, 2006; Rancz et al., 2007). Finally, when the postsynaptic cell is very large and receives only one input, the size of the presynaptic terminal is also big, as are the number of AZs and the recycling pool of SVs. Typical examples are the NMJ (Figure 1C) and the calyx of Held.

Interestingly, even individual neurons can exhibit large differences between neighboring synapses. The hippocampal dentate granule cell mossy fiber, for example, has 11–18 relatively large boutons, each with tens of AZs, and in addition small terminals arising from filopodial extensions of the large ones (Nicoll and Schmitz, 2005).

## Membrane Load During Synaptic Activity

During synaptic activity, vesicles fused at release sites are translocated to the periAZ. This produces a membrane load in this compartment, the magnitude of which depends on the duration and frequency of the stimulation; nevertheless, its relative impact varies with the AZ organization of each terminal. For example, at the mouse NMJ (Figure 1C) from the *levator auris longus* (LAL) muscle, a pure fast muscle, a half-second stimulus train of 50 APs releases about 1700 quanta, the size of readily releasable pool (RRP) of SVs in this terminal (Ruiz et al., 2011). Assuming a mean SV diameter of ~40 nm, the total membrane load is ~8.5 μm^2^ (1700 SVs × πd^2^). However, given the small size of their AZs (0.0054 μm^2^ (60 × 90 nm)); Fukunaga et al., 1983; Fukuoka et al., 1987) and the distance between neighboring AZs) ~0.5 μm; Ruiz et al., 2011), the surface area of each periAZ region (0.5 × 0.5 μm^2^ − 0.0054 μm^2^ = 0.24 μm^2^) increases only by 4.1% when the two primary docked vesicles within each AZ (Nagwaney et al., 2009) fuse.

In another large synapse, the cerebellar mossy fiber bouton, which also has hundreds of release sites (Figure 1B), each one hosting ~7–8 docked vesicles (Xu-Friedman and Regehr, 2004), the mean area of the AZ is about fourfold larger than in mouse motor nerve terminals (0.0216 μm^2^), and the distance between neighboring AZs is ~0.5 μm (Xu-Friedman et al., 2001; Ruiz et al., 2011). Therefore, in this central synapse, if all AZ docked vesicles at rest fuse during phasic nerve activity, the surface area of each periAZ (0.5 × 0.5 μm^2^ − 0.0216 μm^2^ = 0.2284 μm^2^) increases by ~16.5%.

In contrast, in small central synapses (Figure 1A), although the quantal content is much less, a similar number of stimuli may produce a much larger relative increment in the presynaptic membrane surface area. For example, in CA1 excitatory hippocampal presynaptic boutons, which have a mean presynaptic surface area of around 0.2 μm^2^ (Schikorski and Stevens, 1997), and an AZ area of ~0.027 μm^2^, if 10 SVs (the mean size of the RRP) fuse with the presynaptic membrane during 20 Hz, 2 s stimulation, the surface increases by ~0.05 μm^2^, which represents a ~29% increase of the periAZ surface area. Therefore, during high frequency stimulation, if the excess of membrane is not rapidly removed from the AZ and/or the periAZ (Roos and Kelly, 1999) by compensatory endocytosis, or translocated to distant regions for later fission, the relative accumulation of vesicular membrane at the periAZ is larger in small synapses, mainly because their greater number of ready-to-go vesicles per AZ, and their relatively smaller peri-AZ area (Figure 1D).

## When Does Endocytosis Start and How Fast Does it Go?

**Figure 2:**
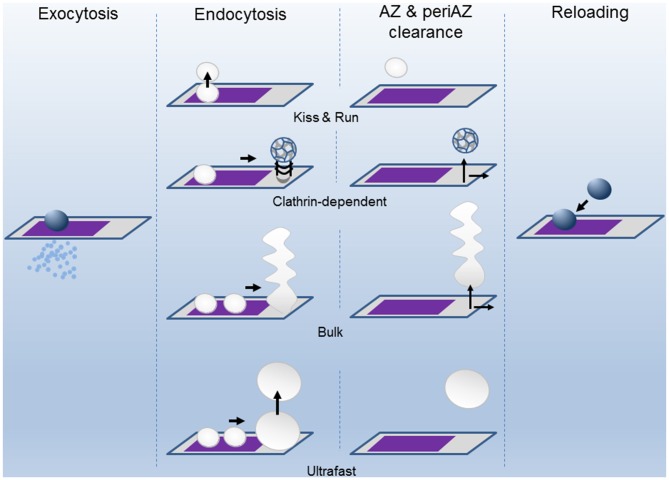
**The successive states of the Release Sites (AZ) and the Peri-AZ during synaptic activity.** Upon calcium entry during the action potential (AP), vesicles fuse at the AZ (purple area) and release neurotransmitter into the synaptic cleft by exocytosis. The SV membrane components are recovered by endocytosis through different pathways: kiss-and-run, clathrin-dependent, bulk, and ultrafast modes. The clearing of the spent vesicular material from AZ and periAZ areas is critical for the subsequent reloading of new vesicles. Clearance can take place at the AZ, at the periAZ (gray area), or outside the periAZ. For simplicity, the shapes of the AZ and periAZ are shown as rectangles and are not drawn to scaled. The widths of the arrows are proportional to the speeds of the process.

Endocytosis is a complex process that has been studied mainly by ultrastructural analysis, electrical capacitance measurements, and by real-time imaging of fluorescent molecules associated with the membranes. These and other techniques have provided evidence that slow and fast modes of endocytosis exist (Figure 2). For example, single SV *clathrin-mediated endocytosis* is a relatively slow process, in the range of tens of seconds (Heuser and Reese, 1973; Granseth et al., 2006; Balaji et al., 2008; Clayton et al., 2008). In the second mode of endocytosis, the so-called *kiss-and-run* mode, after the opening of the fusion pore and releasing of the stored material, the vesicle membrane is rapidly recovered (~1 s; Ceccarelli et al., 1972; Richards et al., 2000, 2005; Aravanis et al., 2003; Gandhi and Stevens, 2003). Also, the retrieval of a large patch of membrane can be achieved at once by what it is called *bulk endocytosis* (Heuser and Reese, 1973; Miller and Heuser, 1984; Holt et al., 2003; Paillart et al., 2003; Clayton et al., 2007; Wu and Wu, 2007; Hayashi et al., 2008). Finally, an ultrafast mode of endocytosis, only active at physiological temperature, has been described in which membrane patches, corresponding to the area of about 4 SVs, are retrieved within 50–100 ms after stimulation (Watanabe et al., 2013a,b, 2014).

Despite the information provided by the different techniques, the mode of vesicle recycling in each synapse type is still controversial. For instance, the *kiss-and-run* mode has been described at the NMJ (Ceccarelli et al., 1973), at the calyx of Held (He et al., 2006), and at hippocampal neurons in culture (Harata et al., 2006). Nevertheless, in small hippocampal boutons, many authors have reported that endocytosis starts with a delay of a few seconds upon stimulation and proceeds slowly. For example, at room temperature, the time constant of endocytosis was estimated to be 14–16 s after 1 AP, and the same up to 100 nerve impulses (Figure 1E; Sankaranarayanan and Ryan, 2001; Mueller et al., 2004; Granseth et al., 2006; Balaji and Ryan, 2007; Balaji et al., 2008). At the adult mouse NMJ, however, the endocytosis time constant, measured *ex vivo*, was reported to be threefold faster (4–5 s after 50 APs; Figure 1E; Tabares et al., 2007; Cano et al., 2012, 2013). When these measurements were done at physiological temperature, the difference persisted between these two synapses. For example, after a single AP the time constant of endocytosis is <2 s at the NMJ (Figure 1E; Gaffield et al., 2009), and between 6–15 s at hippocampal small boutons (Figure 1E; Balaji et al., 2008; Armbruster et al., 2013).

Besides the difference in the prevalent mode of endocytosis in distinct synapses types, differences in the modulation of membrane recycling are also probable. Calcium is a major modulator of endocytosis in small and large synapses. However, depending on the spatiotemporal profile of the calcium increment at release sites, which in turn, depends on the density of calcium channels, the activity of kinases and phosphatases, and the amount and distribution of the different calcium buffers, the outcome may vary. Even more, the modulation process is a very dynamic process in the same synapse. In small hippocampal boutons, the endocytosis kinetics is accelerated when stimulus strength increases from 1 to 25 APs and then progressively slows for stimulus >25–100 APs (Armbruster et al., 2013). One of the mechanisms by which calcium could accelerate endocytosis is the calcineurin-dependent dephosphorylation of the proteins implicated in endocytosis, known as dephosphins, which include dynamin, synaptojanin, amphiphysin, AP-2, AP-180, among others (Marks and McMahon, 1998; Cousin and Robinson, 2000). For example, the amount of dynamin dephosphorylated determines, in turn, the interaction of dynamin with other proteins of the endocytic machine (Koch et al., 2011; Armbruster et al., 2013; Herman and Rosenmund, 2013; Wu et al., 2014). Therefore, the endocytosis speed in different terminals could be regulated not only by the amount of calcium influx during each AP but also by the expression level, and spatial distribution, of the endocytic molecular components in each synapse type.

“*Hot spots*” of endocytic proteins near sites of exocytosis have been described in large synapses, for example, in *Drosophila* (Estes et al., 1996; González-Gaitán and Jäckle, 1997; Roos and Kelly, 1998), snake (Teng et al., 1999) and mouse (Gaffield et al., 2009) NMJs. In central synapses, an enrichment of endocytic proteins at the edges of the AZs is also probable, as suggested by the observation of ultrafast endocytosis in this location at hippocampal boutons (Watanabe et al., 2013b).

The mechanism by which calcium slows endocytosis after prolonged stimulation remains unclear. It could be that calcium increases the rate of endocytosis during stimulation until the capacity of the endocytic machinery becomes insufficient (Sankaranarayanan and Ryan, 2000). At rest, the endocytic machinery is abundant (Roos and Kelly, 1999), but after several rounds of activity, the consumption of the endocytic proteins may slow the process.

The existence of a “*clathrin-coated ready-to-go pool of vesicles*” at rest has been suggested at the frog NMJ (Miller and Heuser, 1984). The origin of these stranded protein spots is, however, not clear. Do they come from fused vesicles that never lost their identity or, alternatively, result after protein intermixing and sorting? A degree of intermixing between fresh and old vesicle proteins has been proposed to occur in hippocampal synapses during phasic stimulation given that stranded, and newly incorporated vesicle proteins are both internalized during compensatory endocytosis (Fernandez-Alfonso et al., 2006; Wienisch and Klingauf, 2006); the longer the stimulus duration, the greater the intermixing. However, it is also possible that endocytosis of both new and stranded protein patches occur in parallel without previous intermixing (Opazo and Rizzoli, 2010). The development of new tools will provide a deeper understanding of the vesicle membrane components dynamics during phasic and sustained stimulation.

## Release Site Reuse

During ongoing synaptic transmission, release sites are repeatedly used. However, has the release site a refractory period after use? Knowing the number of release sites a presynaptic terminal has and the amount of quanta released during a train of stimulation, it is possible to estimate the mean minimum number of times a site is used and the time interval between uses. For example, at the mouse NMJ, a train of 100 APs at 100 Hz, produces the fusion of about 3200 SVs, which represents the size of the whole RRP of vesicles (1700) plus about 1500 more (Ruiz et al., 2011). If evoked release occurs only within the limits of an AZ, each one hosting two release sites (Nagwaney et al., 2009), and all release sites are used at least once, the “*mean reuse index*” is, in this example, 1.88, resulting from dividing cumulative release by the total number of release sites (3200/1700). In this case, 88% of sites release, clear out of vesicular components, dock, prime, and release again in less than 1 s. In the calyx of Held, a similar time of re-usage has been estimated during the first second of stimulation at 100 Hz (Neher, 2010). At higher frequencies of stimulation, the process could be even faster. Such a rapid clearance of the excess of membrane at the release site could be achieved either by endocytosis *in situ* (kiss-and-run) or by moving the vesicular components to the periAZ from where they are later recycled (Figure 2). However, when the membrane load is too large, for example, during sustained high-frequency stimulation, the system becomes less efficient. An excessive membrane accumulation at the periAZ may interfere with the lateral movement of the fused membrane from the AZ to the periAZ, even before depletion of SVs occurs, contributing to short-term depression (Neher, 2010; Hua et al., 2013). Remarkably, translocation, docking, and priming of vesicles during the plateau phase that follows short-term depression can also occur very fast during sustained stimulation. For example, in the cerebellar mossy fiber terminal, this process has been suggested to take 12 ms (Saviane and Silver, 2006), similar to the release site recharging time in some ribbon synapses (Griesinger et al., 2005). These observations suggest that, within a given synapse, not all the release sites has the same capability of being reused at high rates of sustained stimulation. The basis of this heterogeneity is not clear. It could be only apparent if some vesicles fuse outside the “well structured” release sites and this speed up the process (Zenisek et al., 2000; Neher, 2010). Nevertheless, it could also be due to molecular differences in the molecular components involved in docking/priming of the vesicles, to spatial variation in the probability of “*in situ*” endocytosis, and even to disparities in the velocity at which distinct periAZs translocate vesicular components to neighbor regions before endocytosis.

## Summary

The structural and functional properties of presynaptic terminals are principal determinants of the successful transmission of information in the nervous system. Nerve terminals differ not only in size but also in the number, shape and dimensions of their AZs and periAZs, as well as in the magnitude of the recycling pool of SVs. Large terminals have hundreds of AZs and release a large number of quanta in response to stimulation. Small terminals possess one or few AZs and release a low number of quanta. The greater exocytic response in large terminals does not imply a higher load of vesicular components at the periAZs, quite the contrary, the distribution of this material in a larger number of units probably facilitates the management of the membrane excess until endocytosis takes place. The preferred mode of endocytosis used under each regime of activity in each synapse type is still controversial, but it seems to be fast after brief stimulation trains, at least at physiological temperature. With sustained repetitive activation, however, endocytosis becomes slower, probably due, among other factors, to the saturation of the endocytosis mechanisms and the subsequent accumulation of vesicular membrane at the periAZ. Remarkably, many presynaptic terminals can sustain a small and almost constant amount of activity (plateau) upon prolonged high-frequency stimulation, apparently supported by a subpopulation of release sites that can operate faster than others. If the basis of such heterogeneity is at the level of the AZ proteins or the periAZs organization remains to be determined.

## Author Contributions

RC and LT conceived and wrote the manuscript.

## Conflict of Interest Statement

The authors declare that the research was conducted in the absence of any commercial or financial relationships that could be construed as a potential conflict of interest.
